# Choquet integral-based fuzzy molecular characterizations: when global definitions are computed from the dependency among atom/bond contributions (LOVIs/LOEIs)

**DOI:** 10.1186/s13321-018-0306-7

**Published:** 2018-10-25

**Authors:** César R. García-Jacas, Lisset Cabrera-Leyva, Yovani Marrero-Ponce, José Suárez-Lezcano, Fernando Cortés-Guzmán, Mario Pupo-Meriño, Ricardo Vivas-Reyes

**Affiliations:** 1 Instituto de Química, Universidad Nacional Autónoma de México (UNAM), Ciudad de México, México; 2 Grupo de Investigación de Inteligencia Artificial (AIRES), Facultad de Informática, Universidad de Camagüey, Camagüey, Cuba; 3 Grupo de Medicina Molecular y Traslacional (MeM&T), Colegio de Ciencias de la Salud (COCSA), Escuela de Medicina, Edificio de Especialidades Médicas, Universidad San Francisco de Quito (USFQ), Quito, Pichincha Ecuador; 4 Grupo de Investigación Ambiental (GIA), Programas Ambientales, Facultad de Ingenierías, Fundacion Universitaria Tecnologico Comfenalco – Cartagena, Cr 44 DN 30 A, 91, Cartagena, Bolívar Colombia; 5 Pontificia Universidad Católica del Ecuador Sede Esmeraldas (PUCESE), Esmeraldas, Ecuador; 6 Grupo de Investigación de Bioinformática, Universidad de las Ciencias Informáticas (UCI), La Habana, Cuba; 7 Grupo de Química Cuántica y Teórica, Facultad de Ciencias Exactas y Naturales, Programa de Química, Universidad de Cartagena, Campus de San Pablo, Cartagena, Colombia; 8 Grupo CipTec, Facultad de Ingenierias, Fundacion Universitaria Tecnologico Comfenalco – Cartagena, Cr 44 DN 30 A, 91, Cartagena, Bolívar Colombia

**Keywords:** Aggregation operators, Choquet integral, Fuzzy measures, LOVIs, LOEIs, Molecular descriptors, ToMoCoMD-CARDD software, QuBiLS-MIDAS molecular descriptors, QSAR

## Abstract

**Background:**

Several topological (2D) and geometric (3D) molecular descriptors (MDs) are calculated from local vertex/edge invariants (LOVIs/LOEIs) by performing an aggregation process. To this end, norm-, mean- and statistic-based (non-fuzzy) operators are used, under the assumption that LOVIs/LOEIs are independent (orthogonal) values of one another. These operators are based on additive and/or linear measures and, consequently, they cannot be used to encode information from interrelated criteria. Thus, as LOVIs/LOEIs are not orthogonal values, then non-additive (fuzzy) measures can be used to encode the interrelation among them.

**Results:**

General approaches to compute fuzzy 2D/3D-MDs from the contribution of each atom (LOVIs) or covalent bond (LOEIs) within a molecule are proposed, by using the Choquet integral as fuzzy aggregation operator. The Choquet integral-based operator is rather different from the other operators often used for the 2D/3D-MDs calculation. It performs a reordering step to fuse the LOVIs/LOEIs according to their magnitudes and, in addition, it considers the interrelation among them through a fuzzy measure. With this operator, fuzzy definitions can be derived from traditional or recent MDs; for instance, fuzzy Randic-like connectivity indices, fuzzy Balaban-like indices, fuzzy Kier–Hall connectivity indices, among others. To demonstrate the feasibility of using this operator, the QuBiLS-MIDAS 3D-MDs were used as study case and, as a result, a module was built into the corresponding software to compute them (http://tomocomd.com/qubils-midas). Thus, it is the only software reported in the literature that can be employed to determine Choquet integral-based fuzzy MDs. Moreover, regression models were created on eight chemical datasets. In this way, a comparison between the results achieved by the models based on the non-fuzzy QuBiLS-MIDAS 3D-MDs with regard to the ones achieved by the models based on the fuzzy QuBiLS-MIDAS 3D-MDs was made. As a result, the models built with the fuzzy QuBiLS-MIDAS 3D-MDs achieved the best performance, which was statistically corroborated through the Wilcoxon signed-rank test.

**Conclusions:**

All in all, it can be concluded that the Choquet integral constitutes a prominent alternative to compute fuzzy 2D/3D-MDs from LOVIs/LOEIs. In this way, better characterizations of the compounds can be obtained, which will be ultimately useful in enhancing the modelling ability of existing traditional 2D/3D-MDs.

**Electronic supplementary material:**

The online version of this article (10.1186/s13321-018-0306-7) contains supplementary material, which is available to authorized users.

## Introduction

In several application areas, mainly in the multi-criteria decision-making, the information aggregation process is the main step to perform [1–3]. In such a process, the individual criteria are combined into a single value (global criterion), in such a way that all properties contained in each individual criterion are included or reflected in the global criterion, by using an aggregation operator [4, 5]. Thus, several aggregation operators may be used to obtain different global criteria. In this way, decision-makers could consider diversity of criteria with the purpose of making the best final decision. Traditional aggregation operators, where individual criteria are considered as values independent of one another, are those most frequently employed (e.g. OWA-like functions [6–8]). These operators are based on linear and/or additive measures and, thus, they are not suitable to deal with the dependency among criteria.

The dependency or interaction among criteria is an intrinsic feature present in the decision-making tasks in the real world. For example, if various work teams are analyzed to select the one with the best teamwork, and with this purpose the efficiency of each worker belonging to a same team is measured, then the efficiency of each team to do teamwork is not the sum of the individual efficiencies, but the interaction among its workers to achieve the best teamwork. Therefore, it is most suitable to use non-additive measure-based operators, instead of traditional operators, for an approximate modeling of people’s assessment practices. In this sense, the concept of *fuzzy (non*-*additive) measure*, also known as *capacity*, was introduced by Sugeno, in order to model the importance of a coalition within a set of interrelated criteria [9].

According to Lebesgue’s philosophy [10], once a measure is defined, it is possible to obtain an integral with regard to that measure. Thus, associated with the concept of *fuzzy measure*, there is the concept of *fuzzy integral* [11, 12], being the Choquet integral one of the most popular [13–17]. The Choquet integral constitutes a generalization of the Lebesgue integral [10], as well as of other traditional operators (e.g. OWA-like functions), due to the fact that they coincide when the measure used is additive. The Choquet integral has been successfully used in several applications, such as: face recognition [18], rule-based systems [19], data mining [20] and decision-making [21–23]. The success of the Choquet integral as aggregation operator is due to, as already pointed out, its ability of including dependency among criteria by means of a *fuzzy measure* [14, 15].

One of the applications of the aggregation operators is in the chemical structures encoding. This process constitutes an essential step to perform several studies in the cheminformatics field, such as molecular similarity [24] and quantitative structure–activity relationships (QSAR) [25–27]. The codification of chemical structures is performed by means of the molecular descriptors (MDs) calculation. The MDs are values computed from symbolic molecular representations, by applying different mathematical transformations [28] based on a wide variety of theories, such as quantum chemistry [29] and information theory [30]. The MDs are useful values in the sense that they can contribute to obtain a better comprehension on the interpretation of molecular properties, and/or they can integrate a model to predict biological activities in novel compounds [28].

As it can be seen in [28], several procedures to determine MDs are based on the calculation of Local Vertex Invariants (LOVIs) or Local Edge Invariants (LOEIs). These procedures perform an aggregation process on the LOVIs/LOEIs computed to determine the final value (MD) that characterizes the molecular structure (e.g. Randić–Razinger index [31] and local Balaban index [32]). The LOVIs and LOEIs depict each atom (vertex) and covalent bond (edge) of a molecule, respectively. They are computed from graph-based molecular representations without depending on any atom/bond numbering, nor on the rotation and translation of the molecules. LOVIs/LOEIs are represented into *n*-dimensional vectors, where *n* denotes the total number of atoms/bonds. The summation, summation of squares, min and max are the operators often used to obtain global MDs from LOVIs/LOEIs, being the summation operator the one most commonly used.

However, as it has already been pointed out, the use of different aggregation operators yields diversity of global criteria, that is why decision-makers consider several alternatives to make the best final possible decision. Thus, if the MDs whose calculation is based on the aggregation of atom/bond contributions (LOVIs/LOEIs) are determined using several operators, then diversity of global characterizations of the molecules can be computed. For instance, if on the well-known LOVIs called vertex degree, the kurtosis function and the traditional OWA operator are applied, then an aggregation indicating the tailedness of this LOVIs vector, and a weighted aggregation, giving more importance to the vertices (atoms) with the highest degrees, can be calculated, respectively. Both examples are quite different from the common use of the summation operator.

Inspired on this idea, recent strategies to compute topological (2D) and geometric (3D) MDs from atom/bond contributions (LOVIs/LOEIs) have been introduced [33–38]. These 2D/3D-MDs employ aggregation operators based on Minkowski norms (e.g. Euclidean norm), central tendency statistics (e.g. arithmetic mean) and dispersion statistics (e.g. kurtosis). As it has been confirmed elsewhere [39–41], the use of these operators contribute to obtain global 2D/3D-MDs with better information content (variability) and linear independence (orthogonality) than other 2D/3D-MDs reported in the literature. In addition, the diversity of the 2D/3D-MDs computed with these operators allowed to achieve successful outcomes in comparative modeling tasks [42, 43], as well as in several practical applications [44–46].

Nonetheless, up to date, the global 2D/3D-MDs computation from atom/bond contributions (LOVIs/LOEIs) is based on additive operators, under the assumption that these contributions are non-interrelated values. However, it is well-known that the biological activities or properties of the compounds do not only depend on the molecular shape, but also on the interactions that are often non-covalent in nature. Thus, non-additive (fuzzy) measure-based aggregation operators (e.g. Choquet integral) may be used, with the purpose of obtaining an approximate characterization of the interrelation that each atom (or bond) has, regarding the other ones. In this way, 2D/3D-MDs orthogonal to the other existing ones may be obtained, because of the fuzzy basis of their computations.

To the best of our knowledge, only two fuzzy MD types have been introduced: (1) by using pharmacophore-based molecular similarity [47], and (2) by using the number of interposed bonds as the measure of separation among atoms depicting pharmacophore kinds (2D-FPT MDs) [48, 49]. Therefore, fuzzy 2D/3D-MDs computed through an aggregation process on atom/bond contributions (LOVIs/LOEIs) have not been reported to date. Consequently, this work is aimed at introducing a different way for the global 2D/3D-MDs computation from LOVIs/LOEIs, by using the Choquet integral as fuzzy aggregation operator. This report is planned as follows. Second section defines some concepts regarding the fuzzy measures and the Choquet integral. Third section presents the adaptation of several procedures to compute fuzzy MDs. Fourth section presents a practical example. Fifth section studies the feasibility of using this approach. Last section describes the main findings and conclusions.

## Background of fuzzy measures and Choquet integral

### Definition of fuzzy measure and singleton measure. L_mδ_-measure: fuzzy measure composed of maximized L-measure and Delta-measure

The fuzzy measures (or capacities) are functions that determine a weight considering the interrelation (or dependency) among criteria within a subset [11, 14]. Formally, let a universal set $X = \left\{ {x_{1} ,x_{2} , \ldots ,x_{N} } \right\}$ and $P\left( X \right)$ be the power set of $X$, $P\left( X \right) = 2^{N}$, then a fuzzy measure [9] or capacity [13] on $X$ is a set function $\mu$: $P\left( X \right) \to \left[ {0,\,1} \right]$ that fulfils the following axioms:

1. $\mu \left( \emptyset \right) = 0$ (lower boundary condition).
2. $\mu \left( X \right) = 1$ (upper boundary condition).
3. $$If\,A,\,B\, \in \,P\left( X \right)\, \wedge \,A\, \subseteq B\, \Rightarrow \,\mu \left( A \right) \le \mu \left( B \right)$$ (monotonicity).

Therefore, for any $A \subseteq X$, $\mu \left( A \right)$ can be considered as the degree of importance (or weight) of the combination $A$ of criteria. If $\left| A \right| = 1$, then $\mu \left( A \right) = \mu \left( {x_{i} } \right)$, and it constitutes the traditional weight when element $x_{i}$ is considered separately. It is important to highlight that $\mu \left( {x_{i} } \right)$ is denominated as fuzzy density or singleton measure, denoted as $s\left( {x_{i} } \right)$, when any $A \subseteq X$ has a single element $x_{i}$. Moreover, a fuzzy measure is additive if $\mu \left( {A \cup B} \right) = \,\mu \left( A \right) + \mu \left( B \right)$, whenever $A \cap B = \emptyset$. Thus, it is enough to determine $\forall \,x_{i} \in X$ the corresponding $s\left( {x_{i} } \right)$ to define the measure completely. Other important properties of the fuzzy measures are the superadditivity and subadditivity. The former indicates high synergy or cooperative action among the criteria of a set, while the latter expresses the opposite. So, the additivity can be interpreted as the no interaction among the criteria of a set.

Several fuzzy measures have been reported in the literature, such as the Sugeno λ-measure [9] (that was the first one proposed), the P-measure [50], the Shapley values [51], the *k*-order fuzzy measure [52], among others [53–55]. The λ-measure and P-measure are among the most widely employed. The λ-measure is not a closed form [9], whereas the P-measure is not sensitive enough, because it only determines the max value of the input set [50] (see Additional file 1). In addition, when the number of criteria is large, then the computation of the λ parameter is quite complex in the λ-measure, because a polynomial equation of higher order must be resolved. In order to tackle these drawbacks, a fuzzy measure comprised of the Maximized L-measure (L_m_-measure) [56, 57] and Delta-measure (δ-measure) [58], denoted as L_mδ_-measure, was proposed by Liu et al. [54, 59].

Formally, a fuzzy L_mδ_-measure, $g_{{L_{m\delta } }}$, on finite set $X = \left\{ {x_{1} ,x_{2} , \ldots ,x_{N} } \right\}$ is defined as follows:

1 $$g_{{L_{m\delta } }} (A) = \left\{ {\begin{array}{*{20}l} {\mathop {\hbox{max} }\nolimits_{x \in A} s(x)} \hfill & {L = - 1} \hfill \\ {\frac{{\left( {1 + L} \right)\sum\nolimits_{x \in A} {s\left( x \right)} \left[ {1 + L\mathop {\hbox{max} }\nolimits_{x \in A} s\left( x \right)} \right]}}{{1 + L\sum\nolimits_{x \in A} {s\left( x \right)} }} - L\mathop {\hbox{max} }\nolimits_{x \in A} s\left( x \right)} \hfill & {L \in \left( { - 1,0} \right]} \hfill \\ {\frac{{L\left( {\left| A \right| - 1} \right)\sum\nolimits_{x \in A} {s\left( x \right)} \left[ {1 - \sum\nolimits_{x \in A} {s\left( x \right)} } \right]}}{{\left( {n - \left| A \right|} \right)\sum\nolimits_{x \in X - A} {s\left( x \right) + L\left( {\left| A \right| - 1} \right)\sum\nolimits_{x \in A} {s\left( x \right)} } }} + \sum\nolimits_{x \in A} {s\left( x \right)} } \hfill & {L \in \left( {0,\infty } \right)} \hfill \\ \end{array} } \right.$$

where $A \subseteq X$, $L \in \left[ { - 1,\,\infty } \right)$, $s\left( \cdot \right)$ is a singleton measure for each $x_{i} \in X$, $\sum\nolimits_{x \in X} {s(x)} = 1$, $g_{{L_{m\delta } }} \left( \emptyset \right) = 0$ and $g_{{L_{m\delta } }} \left( X \right) = 1$. This fuzzy measure satisfies the following properties: (1) L_mδ_-measure is an increasing function on $L$; (2) if $L = - 1$, then L_mδ_-measure is just the P-measure; (3) if $L = 0$, then L_mδ_-measure is additive (it coincides with the λ-measure when $\lambda = 0$—see Additional file 1); (4) if $- 1 < L < 0$, then L_mδ_-measure satisfies the subadditivity property (low synergism); and (5) if $0 < L < \infty$, then L_mδ_-measure satisfies the superadditivity property (high synergism).

### Mathematical definition of the Choquet integral

The Choquet integral was first presented in capacity theory [13]. Its use as an integral with regard to fuzzy measures was then introduced by Hohle [60] and, it was later rediscovered by Murofushi and Sugeno [61, 62]. This integral, as an n-place operator, has been used in several works [18–23], in order to fuse information when interrelated criteria are accounted for. Formally, let a finite set $X = \left\{ {x_{1} ,\,x_{2} ,\, \ldots ,\,x_{N} } \right\}|X \in \Re_{ \ge 0}^{N}$ and $\mu$ be a fuzzy measure on $N$, then the Choquet integral of $X$ with respect to $\mu$ is a function $C_{\mu }$: $\Re_{ \ge 0}^{N} \to \Re_{ \ge 0}$ according to the next expression:

2 $$C_{\mu } \left( {x_{1} ,x_{2} , \ldots ,x_{N} } \right) = \sum\limits_{i = 1}^{N} {x_{\left( i \right)} \left[ {\mu \left( {A_{\left( i \right)} } \right) - \mu \left( {A_{{\left( {i - 1} \right)}} } \right)} \right]}$$

where $\left( \cdot \right)$ denotes a permutation on $N$, so that $x_{\left( 1 \right)} \ge x_{\left( 2 \right)} \ge \cdots \ge x_{\left( N \right)}$. That is, $x_{\left( i \right)}$ is the *i*-*th* largest value in the set $\left\{ {x_{1} ,x_{2} , \ldots ,x_{N} } \right\}$. Thus, $A_{\left( i \right)} = \left\{ {x_{\left( i \right)} , \ldots ,x_{\left( N \right)} } \right\}$ when $i \ge 1$, and $A_{0} = \emptyset$. So, for instance, if $X = \left\{ {x_{1} ,\,x_{2} ,\,x_{3} } \right\}|x_{2} \ge x_{3} \ge x_{1}$, then the Choquet integral-based aggregation is computed as follows:

$$\begin{aligned} C_{\mu } \left( {x_{1} ,x_{2} ,x_{3} } \right) & = x_{2} \left[ {\mu \left( {x_{2} ,x_{3} ,x_{1} } \right) - \mu \left( {x_{3} ,x_{1} } \right)} \right] \\ & \quad + x_{3} \left[ {\mu \left( {x_{3} ,x_{1} } \right) - \mu \left( {x_{1} } \right)} \right] \\ & \quad + x_{1} \left[ {\mu \left( {x_{1} } \right)} \right] \\ \end{aligned}$$

As it can be seen, this operator performs a reordering of its arguments according to their magnitudes, as the OWA-like operators do [6, 7]. Indeed, as it has been demonstrated elsewhere [15, 63, 64], the Choquet integral constitutes a generalization of the latter. Moreover, it can be observed that since the values are ordered in decreasing order, then $\mu \left( {A_{\left( i \right)} } \right) \ge \mu \left( {A_{{\left( {i - 1} \right)}} } \right)$. Lastly, it is important to highlight that the Choquet integral fulfils some properties, such as: (1) it is a continuous function; (2) it is homogeneous of degree 1; (3) it is monotonic and idempotent, if and only if $\mu$ is a fuzzy measure; and (4) it is compensative when $\mu$ is a normalized fuzzy measure.

## Extending traditional functions to derive Choquet integral-based fuzzy descriptors

Table G3 in [28] shows several traditional functions to derive classic 2D/3D-MDs from atom/bond contributions (LOVIs/LOEIs), e.g. the Zagreb indices [65], the Balaban-like indices [66], the Wiener-type indices [67], among others [28]. As it can be observed, these functions are mainly based on the summation and product aggregation operators. Consequently, the functions described in Table G3 do not consider the possible interrelation among LOVIs/LOEIs, which are only aggregated of linear and/or additive ways. Therefore, in order to consider the dependency among LOVIs/LOEIs, some of these traditional functions can be extended to compute fuzzy 2D/3D-MDs (FMDs), by using the Choquet integral $\left( {C_{\mu } } \right)$ with respect to a fuzzy measure $\mu$ as shown below:

3 $$MD^{1} \left( {L,\alpha^{'} ,\lambda^{'} } \right) = \alpha^{'} \cdot \sum\limits_{i = 1}^{A} {L_{i}^{{\lambda {\prime }}} } \to FMD_{{C_{\mu } }}^{1} \left( {L,\alpha^{'} ,\lambda^{'} } \right) = C_{\mu } \left( {\alpha^{'} \cdot L_{1}^{{\lambda {\prime }}} , \ldots ,\alpha^{'} \cdot L_{A}^{{\lambda {\prime }}} } \right)$$

4 $$\begin{aligned} MD^{4} \left( {L,\alpha^{'} ,\lambda^{'} } \right) & = \alpha^{'} \sum\limits_{i = 1}^{A} {\sum\limits_{j = 1}^{A} {a_{ij} \left( {L_{i} \cdot L_{j} } \right)^{{\lambda^{'} }} } } \to FMD_{{C_{\mu } }}^{4} \left( {L,\alpha^{'} ,\lambda^{'} } \right) = C_{\mu } \left( {L_{1} , \ldots ,L_{W} } \right) \\ & \quad |L_{w} = a_{ij} \cdot \alpha^{'} \cdot \left( {L_{i} \cdot L_{j} } \right)^{{\lambda {\prime }}} \quad \forall i,j \in V \\ \end{aligned}$$

5 $$\begin{aligned} MD^{5} \left( {L,\alpha^{'} ,\lambda^{'} } \right) & = \alpha^{'} \sum\limits_{i = 1}^{A} {\sum\limits_{j = 1}^{A} {\left( {L_{i} \cdot L_{j} } \right)^{{\lambda^{'} }} } } \to FMD_{{C_{\mu } }}^{5} \left( {L,\alpha^{'} ,\lambda^{'} } \right) = C_{\mu } \left( {L_{1} , \ldots ,L_{W} } \right) \\ & \quad |L_{w} = \alpha^{'} \cdot \left( {L_{i} \cdot L_{j} } \right)^{{\lambda {\prime }}} \quad \forall i,j \in V,i \ne j \\ \end{aligned}$$

6 $$MD^{6} \left( {L,\alpha^{'} ,\lambda^{'} } \right) = \alpha^{'} \sum\limits_{k = 1}^{K} {\left( {\prod\limits_{i = 1}^{{n_{k} }} {L_{i} } } \right)}_{k}^{{\lambda^{'} }} \to FMD_{{C_{\mu } }}^{6} \left( {L,\alpha^{'} ,\lambda^{'} } \right) = \alpha^{'} \sum\limits_{k = 1}^{K} {C_{\mu } \left( {L_{1}^{{\lambda^{'} }} , \ldots ,L_{{n_{k} }}^{{\lambda {\prime }}} } \right)_{k} }$$

7 $$\begin{aligned} MD^{7} \left( {L,\alpha^{'} ,\lambda^{'} ,k} \right) & = \alpha^{'} \sum\limits_{i \in k} {\sum\limits_{j \in k} {a_{ij} \left( {L_{i} \cdot L_{j} } \right)^{{\lambda^{'} }} } } \to FMD_{{C_{\mu } }}^{7} \left( {L,\alpha^{'} ,\lambda^{'} ,k} \right) = C_{\mu } \left( {L_{1} , \ldots ,L_{W} } \right) \\ & \quad |L_{w} = a_{ij} \cdot \alpha^{'} \cdot \left( {L_{i} \cdot L_{j} } \right)^{{\lambda {\prime }}} \quad \forall i,j \in k \\ \end{aligned}$$

where $L_{i}$ and $L_{j}$ are the LOVI values for any pair vertices $v_{i}$ and $v_{j}$ (atoms) of a molecular graph $G$, $A$ is the number of vertices, $V$ represents the set of vertices of $G$, $a_{ij}$ denotes the coefficients of the adjacency matrix of $G$ (1 for adjacent vertices, 0 otherwise), $K$ is the total number of graph fragments to be considered, $n_{k}$ is the number of vertices within the kth fragment, and $\alpha^{'}$ and $\lambda^{'}$ are two real parameters. The superscript in notation $MD$ represents the numbering used to identify these functions in Table G3 in [28]. Accordingly, this numbering is also used to identify the corresponding fuzzy formulations $\left( {FMD_{{C_{\mu } }} } \right)$. Note that these definitions can also be used to compute fuzzy 2D/3D-MDs from LOEIs in place of LOVIs.

From these fuzzy formulations, several specific descriptors can be computed, for instance: (1) from $$FMD_{{C_{\mu } }}^{1}$$ for $$\alpha^{'} = \lambda^{'} = 1$$, fuzzy DIVATI MDs [41], fuzzy GT-STAF MDs [34] and fuzzy QuBiLS-MAS MDs [37] can be obtained, when the LOVIs vector is computed with some of those families; (2) if vector $$L$$ is computed with the vertex degree invariant, then from $$FMD_{{C_{\mu } }}^{1}$$ for $$\alpha^{'} = 1$$ and $$\lambda^{'} = 2$$, from $$FMD_{{C_{\mu } }}^{4}$$ for $$\alpha^{'} = \lambda^{'} = 1$$ and from $$FMD_{{C_{\mu } }}^{4}$$ for $$\alpha^{'} = 1$$ and $$\lambda^{'} = - {\raise0.7ex\hbox{$1$} \!\mathord{\left/ {\vphantom {1 2}}\right.\kern-0pt} \!\lower0.7ex\hbox{$2$}}$$, the fuzzy first Zagreb index [65], the fuzzy second Zagreb index [65] and the fuzzy Randic connectivity index [68] can be obtained, respectively; (3) from $$FMD_{{C_{\mu } }}^{4}$$ for $$\alpha^{'} = \frac{B}{C + 1}$$ (B is the number of graph edges (covalent edges) and C is the number of rings) and $$\lambda^{'} = - {\raise0.7ex\hbox{$1$} \!\mathord{\left/ {\vphantom {1 2}}\right.\kern-0pt} \!\lower0.7ex\hbox{$2$}}$$, the fuzzy Balaban-like indices can be determined [32]; (4) from $$FMD_{{C_{\mu } }}^{6}$$ for $$\alpha^{'} = 1$$ and $$\lambda^{'} = - {\raise0.7ex\hbox{$1$} \!\mathord{\left/ {\vphantom {1 2}}\right.\kern-0pt} \!\lower0.7ex\hbox{$2$}}$$, the fuzzy Kier–Hall connectivity indices can be obtained [69]; and (5) from $$FMD_{{C_{\mu } }}^{7}$$, fuzzy autocorrelation MDs can be computed. A practical example is presented below.

## Practical example: Choquet integral-based fuzzy QuBiLS-MIDAS molecular descriptors

Geometric multi-linear algebraic MDs, also known as QuBiLS-MIDAS, were introduced as a novel framework to characterize molecular structures [39, 40]. These 3D-MDs are the only ones that encode structural information between two atoms of a molecule using several metrics (e.g. Soergel) [39], as well as chemical information corresponding to the relations between three and four atoms through multi-metrics (e.g. bond and dihedral angle) [40]. QSAR studies on eight benchmark chemical datasets were carried out [43], where the QuBiLS-MIDAS 3D-MDs yielded significantly superior outcomes with respect to 12 2D/3D-QSAR methodologies established in the literature. The QuBiLS-MIDAS 3D-MDs were also applied in the prediction of inhibitory activity of bromodomain modulators (BRD2, BRD3 and BRD4) with successful results [46].

### Traditional (no fuzzy) definition of the QuBiLS-MIDAS descriptors

The traditional QuBiLS-MIDAS MDs are computed from atom-level descriptors (LOVIs). Thus, the *k*-*th atom*-*level two*-*linear* $\left[ {{}_{{ns\left( {ss,ds,mp} \right)}}^{{\left( * \right)\left( {NQ} \right)}} b_{\left( F \right)}^{a,k} \left( {\bar{x},\bar{y}} \right)} \right]$, *three*-*linear* $\left[ {{}_{{ns\left( {ss,mp} \right)}}^{{\left( * \right)\left( {NQ} \right)}} tr_{\left( F \right)}^{a,k} \left( {\bar{x},\bar{y},\bar{z}} \right)} \right]$ and *four*-*linear* $\left[ {{}_{{ns\left( {ss,mp} \right)}}^{{\left( * \right)\left( {NQ} \right)}} qu_{\left( F \right)}^{a,k} \left( {\bar{x},\bar{y},\bar{z},\bar{w}} \right)} \right]$ QuBiLS-MIDAS 3D-MDs are calculated as *N*-linear (multi-linear) algebraic maps in ${\mathbb{R}}^{n}$, in a canonical basis set, when geometric coordinate-based relations among two $\left( {N = 2} \right)$, three $\left( {N = 3} \right)$ and four $\left( {N = 4} \right)$ atoms are considered, respectively [39, 40]. The formulation (indicial notation) of these 3D-MDs is as follows:

8 $${}_{{{}_{{ns\left( {ss,ds,mp} \right)}}^{{}} b\left( F \right)}}^{{\left( * \right)\left( {NQ} \right)}} L_{a}^{k} = {}_{{ns\left( {ss,ds,mp} \right)}}^{{\left( * \right)\left( {NQ} \right)}} b_{\left( F \right)}^{a,k} \left( {\bar{x},\bar{y}} \right) = {}_{{ns\left( {ss,ds,mp} \right)}}^{{}} \left( {{{\mathbb{N}}{\mathbb{Q}}}} \right){\mathbb{G}}_{ij\left( F \right)}^{a,k} x^{i\left( * \right)} y^{j\left( * \right)}$$

9 $${}_{{{}_{{ns\left( {ss,mp} \right)}}^{{}} tr\left( F \right)}}^{{\left( * \right)\left( {NQ} \right)}} L_{a}^{k} = {}_{{ns\left( {ss,mp} \right)}}^{{\left( * \right)\left( {NQ} \right)}} tr_{\left( F \right)}^{a,k} \left( {\bar{x},\bar{y},\bar{z}} \right) = {}_{{ns\left( {ss,mp} \right)}}^{{}} \left( {{{\mathbb{N}}{\mathbb{Q}}}} \right){{\mathbb{G}}{\mathbb{T}}}_{ijl\left( F \right)}^{a,k} x^{i\left( * \right)} y^{j\left( * \right)} z^{l\left( * \right)}$$

10 $${}_{{{}_{{ns\left( {ss,mp} \right)}}^{{}} qu\left( F \right)}}^{{\left( * \right)\left( {NQ} \right)}} L_{a}^{k} = {}_{{ns\left( {ss,mp} \right)}}^{{\left( * \right)\left( {NQ} \right)}} qu_{\left( F \right)}^{a,k} \left( {\bar{x},\bar{y},\bar{z},\bar{w}} \right) = {}_{{ns\left( {ss,mp} \right)}}^{{}} \left( {{{\mathbb{N}}{\mathbb{Q}}}} \right){{\mathbb{G}}{\mathbb{Q}}}_{ijlh\left( F \right)}^{a,k} x^{i\left( * \right)} y^{j\left( * \right)} z^{l\left( * \right)} w^{h\left( * \right)}$$

where *n* is the number of atoms, “*a*” is a particular atom $\left( {a = 1, \ldots ,n} \right)$, the indices $i,j,l,h =$ $1 \ldots n$ denote the entries of the matrices and property vectors, $k = \pm 1,\, \ldots ,\, \pm 12$ is the power of the matrices, and $x^{1\left( * \right)} ,\, \ldots ,\,x^{n\left( * \right)}$, $y^{1\left( * \right)} ,\, \ldots ,\,y^{n\left( * \right)}$, $z^{1\left( * \right)} ,\, \ldots ,\,z^{n\left( * \right)}$ and $w^{1\left( * \right)} ,\, \ldots ,\,w^{n\left( * \right)}$ are the coefficients of the property vectors $\overline{{x^{\left( * \right)} }}$, $\overline{{y^{\left( * \right)} }}$, $\overline{{z^{\left( * \right)} }}$ and $\overline{{w^{\left( * \right)} }}$, respectively, when central chirality aspects are codified (*) or not [70]). Moreover, ${}_{{ns\left( {ss,ds,mp} \right)}}^{{}} \left( {{{\mathbb{N}}{\mathbb{Q}}}} \right){\mathbb{G}}_{\left( F \right)}^{a,k}$, ${}_{{ns\left( {ss,mp} \right)}}^{{}} \left( {{{\mathbb{N}}{\mathbb{Q}}}} \right){{\mathbb{G}}{\mathbb{T}}}_{\left( F \right)}^{a,k}$ and ${}_{{ns\left( {ss,mp} \right)}}^{{}} \left( {{{\mathbb{N}}{\mathbb{Q}}}} \right){{\mathbb{G}}{\mathbb{Q}}}_{\left( F \right)}^{a,k}$ denote the two-, three- and four-tuple atom-level matrices for each atom “*a*”, respectively. From these atom-level matrices, then atom-level descriptors (LOVI) are determined. Each LOVI constitutes an entry $\left( {L_{a} } \right)$ in the corresponding vector of atom-level descriptors $L^{k}$ (LOVIs vector). The notations (${{\mathbb{N}}{\mathbb{Q}}}$, *F*, *ns*, *ss*, *ds* and *mp*) between parentheses are not mandatory during the calculation and they will be explained below.

*Keep*-*all total matrices* (${\mathbb{G}}^{k}$, ${{\mathbb{G}}{\mathbb{T}}}^{k}$ and ${{\mathbb{G}}{\mathbb{Q}}}^{k}$) are the basis to compute these 3D-MDs. For $k = 1$, the entries of the matrices ${\mathbb{G}}^{1}$, ${{\mathbb{G}}{\mathbb{T}}}^{1}$ and ${{\mathbb{G}}{\mathbb{Q}}}^{1}$ denote the information encoded for the relations between two, three and four atoms of a molecule, respectively, by using several metrics and multi-metrics (see Tables 1–2 in [43]). From these matrices, *neighborhood*-*quotient matrices* (${{\mathbb{N}}{\mathbb{Q}}{\mathbb{G}}}^{k}$, ${{\mathbb{N}}{\mathbb{Q}}{\mathbb{G}}{\mathbb{T}}}^{k}$ and ${{\mathbb{N}}{\mathbb{Q}}{\mathbb{G}}{\mathbb{Q}}}^{k}$) may be obtained, which contain information of the inter-atomic relations that satisfy certain molecular cutoffs [71]. *Local*-*fragment matrices* (${\mathbb{G}}_{F}^{k}$, ${{\mathbb{G}}{\mathbb{T}}}_{F}^{k}$ and ${{\mathbb{G}}{\mathbb{Q}}}_{F}^{k}$) may also be computed (see Equation 13 in [39] and Equations 17–18 in [40]) to encode information of chemical fragments or atom-types (*F*) of interest. Normalized matrices may also be obtained using the *simple*-*stochastic* (*ss*—see Equation 10 in [39] and Equations 13–14 in [40]), *double*-*stochastic* (*ds*) [72] and *mutual probability* (*mp*—see Equation 12 in [39] and Equation 15–16 in [40]) procedures. If no normalization procedure is used, then the matrices are *non*-*stochastic* (*ns*).

Finally, from the *keep*-*all* (neighborhood-quotient) *non*-*stochastic* (simple-stochastic, double-stochastic or mutual-probability) *total* (local-fragment) *matrices* [${}_{{ns\left( {ss,ds,mp} \right)}}^{{}} \left( {{{\mathbb{N}}{\mathbb{Q}}}} \right){\mathbb{G}}_{\left( F \right)}^{k}$, ${}_{{ns\left( {ss,mp} \right)}}^{{}} \left( {{{\mathbb{N}}{\mathbb{Q}}}} \right){{\mathbb{G}}{\mathbb{T}}}_{\left( F \right)}^{k}$ and ${}_{{ns\left( {ss,mp} \right)}}^{{}} \left( {{{\mathbb{N}}{\mathbb{Q}}}} \right){{\mathbb{G}}{\mathbb{Q}}}_{\left( F \right)}^{k}$], the respective atom-level matrices are calculated (see Equation 9 in [39] and Equations 3–4 in [40]) with the purpose of determining the vectors of LOVIs (see Eqs. 8–10). After that, and considering the atom-level descriptors (LOVIs) as independent values of one another, then the (non-fuzzy) *global k*-*th two*-*linear*, *three*-*linear* and *four*-*linear* QuBiLS-MIDAS 3D-MDs are obtained using one or several (non-fuzzy) aggregation operators based on the Minkowski definition (e.g. Euclidean norm), central tendency statistics (e.g. harmonic mean) and dispersion statistics (e.g. variance) [39, 40].

### Fuzzy definition of the QuBiLS-MIDAS descriptors based on the Choquet integral

**Scheme 1:**
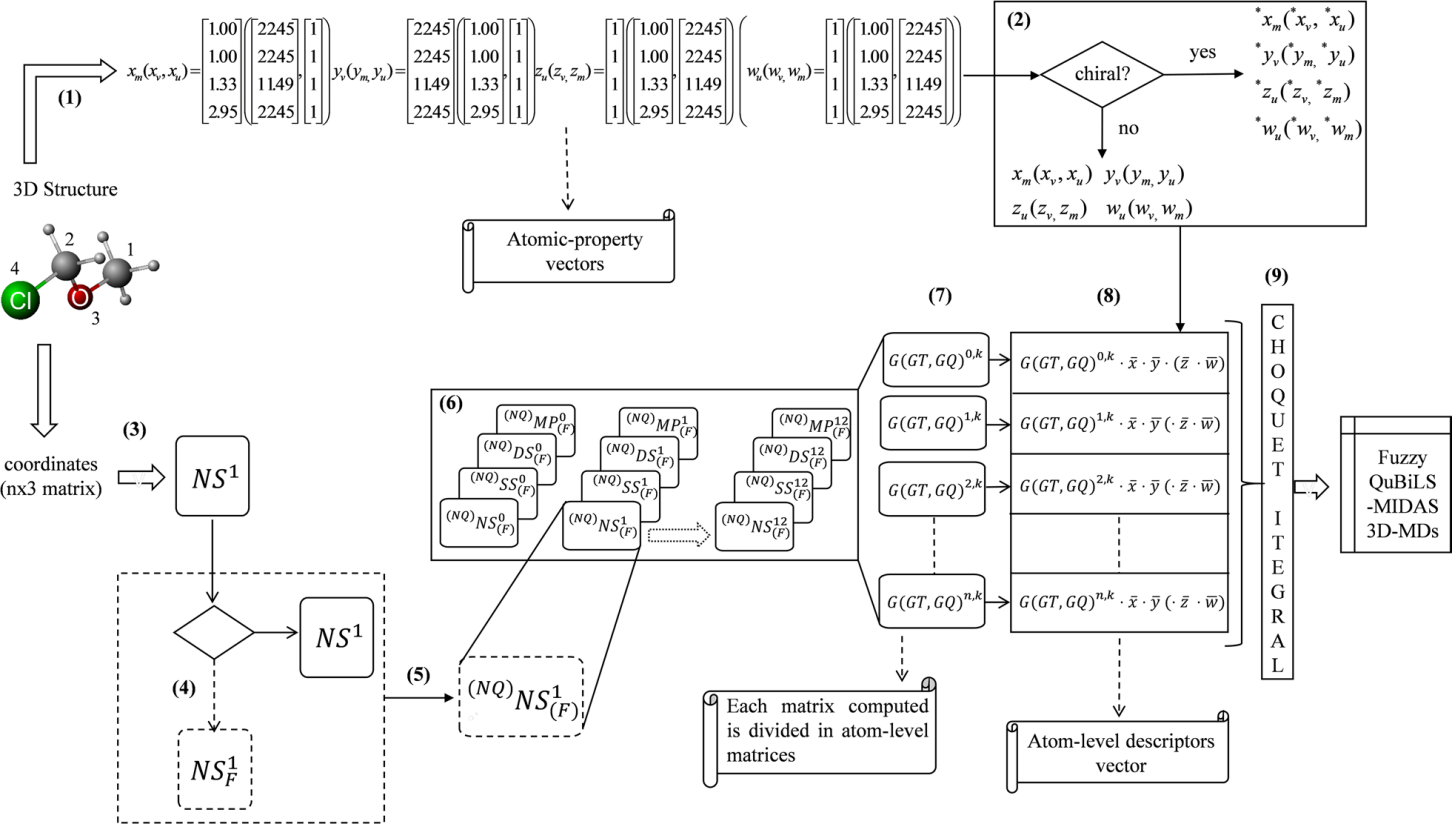
General workflow for the calculation of the Choquet integral-based fuzzy QuBiLS-MIDAS molecular descriptors. (1) Computation of the molecular vectors according to the atomic properties selected; (2) Computation of the molecular vectors considering chiral properties (optional); (3) Computation of the non-stochastic two-tuple, three-tuple or four-tuple matrices, for $k = 1$, from 3D Cartesian coordinates of each atom; (4) Consider atom-types or local-fragments (optional); (5) Apply molecular cutoffs (optional); (6) Computation of the simple-stochastic, double-stochastic and mutual probability matrices, as well as of the *k*th matrices using the Hadamard product; (7) Split the matrices calculated in atom-level matrices; (8) Computation of the atom-level indices (descriptors) using the molecular vectors calculated in the steps 1–2; and (9) Apply the Choquet integral on the vector of atom-level descriptors

So far, QuBiLS-MIDAS 3D-MDs are computed from LOVIs considered as non-interrelated values. However, as already pointed out, the biological activities or properties of the compounds do not only depend on the molecular shape, but also on the interactions that are often non-covalent in nature. Therefore, the interrelation among atomic contributions (LOVIs) may be an aspect to consider during molecular encoding. In this way, from the corresponding LOVIs vector and considering their coefficients as interrelated values of one another, then the *fuzzy global k*-*th two*-*linear, three*-*linear and four*-*linear QuBiLS*-*MIDAS 3D*-*MDs* are computed using the definition of Choquet integral (see Eq. 2) as shown below:

11 $${}_{{ns\left( {ss,ds,mp} \right)}}^{{\left( * \right)\left( {NQ} \right)}} b_{\left( F \right)}^{k} \left( {\bar{x},\bar{y}} \right) = C_{\mu } \left( {{}_{{{}_{{ns\left( {ss,ds,mp} \right)}}^{{}} b\left( F \right)}}^{{\left( * \right)\left( {NQ} \right)}} L_{1}^{k} , \ldots ,{}_{{{}_{{ns\left( {ss,ds,mp} \right)}}^{{}} b\left( F \right)}}^{{\left( * \right)\left( {NQ} \right)}} L_{n}^{k} } \right)$$

12 $${}_{{ns\left( {ss,mp} \right)}}^{{\left( * \right)\left( {NQ} \right)}} tr_{\left( F \right)}^{k} \left( {\bar{x},\bar{y},\bar{z}} \right) = C_{\mu } \left( {{}_{{{}_{{ns\left( {ss,mp} \right)}}^{{}} tr\left( F \right)}}^{{\left( * \right)\left( {NQ} \right)}} L_{1}^{k} , \ldots ,{}_{{{}_{{ns\left( {ss,mp} \right)}}^{{}} tr\left( F \right)}}^{{\left( * \right)\left( {NQ} \right)}} L_{n}^{k} } \right)$$

13 $${}_{{ns\left( {ss,mp} \right)}}^{{\left( * \right)\left( {NQ} \right)}} qu_{\left( F \right)}^{k} \left( {\bar{x},\bar{y},\bar{z},\bar{w}} \right) = C_{\mu } \left( {{}_{{{}_{{ns\left( {ss,mp} \right)}}^{{}} qu\left( F \right)}}^{{\left( * \right)\left( {NQ} \right)}} L_{1}^{k} , \ldots ,{}_{{{}_{{ns\left( {ss,mp} \right)}}^{{}} qu\left( F \right)}}^{{\left( * \right)\left( {NQ} \right)}} L_{n}^{k} } \right)$$

where $C_{\mu } ( \ldots )$ is the Choquet integral with respect to a fuzzy measure $\mu$; and ${}_{{{}_{{ns\left( {ss,ds,mp} \right)}}^{{}} b\left( F \right)}}^{{\left( * \right)\left( {NQ} \right)}} L_{a}^{k}$, ${}_{{{}_{{ns\left( {ss,mp} \right)}}^{{}} tr\left( F \right)}}^{{\left( * \right)\left( {NQ} \right)}} L_{a}^{k}$ and ${}_{{{}_{{ns\left( {ss,mp} \right)}}^{{}} qu\left( F \right)}}^{{\left( * \right)\left( {NQ} \right)}} L_{a}^{k}$ are the kth two-linear, three-linear and four-linear atom-level descriptors (LOVIs), respectively, determined for each atom “*a*” of a molecule, according to Eqs. 8–10. Note that these formulations coincide with the definition $FMD_{{C_{\mu } }}^{1}$ for $\alpha^{'} = \lambda^{'} = 1$ (see Eq. 3). Scheme 1 shows a flowchart regarding the calculation of these fuzzy 3D-MDs.

The L_mδ_-measure [54, 59] (see Eq. 1) is used to compute the importance (weight) of the interrelation among atom-level descriptors (LOVIs) during the fuzzy QuBiLS-MIDAS 3D-MDs calculation. As it can be seen in Eq. 1, the L_mδ_-measure depends on a singleton measure $s\left( x \right)$, so that $\sum\nolimits_{x \in X} {s(x)} = 1$, being $X$ a finite set of elements. In this case, set $X$ is the LOVIs vector $\left( {L^{k} } \right)$ computed for a compound, either the kth two-linear, three-linear or four-linear atom-level QuBiLS-MIDAS 3D-MDs. Therefore, the singleton measure $s\left( {L_{a}^{k} } \right)$, $\forall \,L_{a}^{k} \in L^{k}$, determines the belonging degree of the descriptor for atom “*a*” ($L_{a}^{k}$—see Eqs. 8–10) within the set of atom-level descriptors $\left( {L^{k} } \right)$. To this end, the following two functions [73] were used:

Aggregated Objects Type 1 (AO1):

14 $$s(L_{i}^{k} ) = \frac{{b_{i}^{\alpha } }}{{\sum\nolimits_{i = 1}^{n} {b_{i}^{\alpha } } }},\quad i = 1,2, \ldots ,n$$

**Scheme 2:**
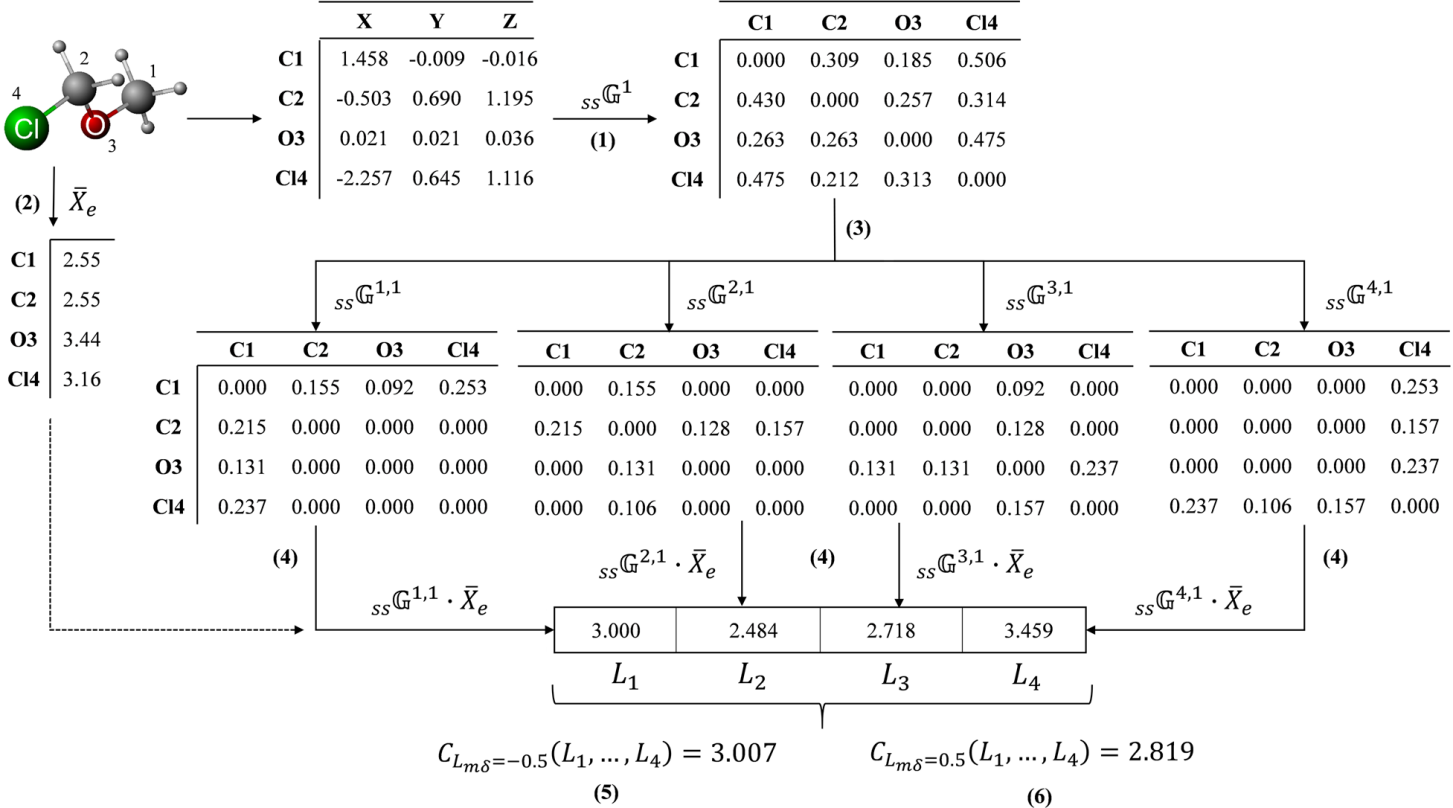
Workflow for the calculation of a specific fuzzy two-linear descriptor based on the linear algebraic form, Euclidean metric, simple-stochastic matrix, electronegativity as property, and the Choquet integral as aggregation operator. (1) Computation of the simple-stochastic matrix for $k = 1$ $\left( {{}_{ss}^{{}} {\mathbb{G}}^{1} } \right)$ from the 3D coordinates matrix, by using the Euclidean metric; (2) Computation of the property vector using the electronegativity property, $\bar{X}_{e}$; (3) Split the ${}_{ss}^{{}} {\mathbb{G}}^{1}$ matrix into “n” (number of atoms) atom-level matrices, ${}_{ss}^{{}} {\mathbb{G}}^{a,1}$, where “a” represent a specific atom; (4) Computation of the atom-level descriptors, by multiplying each ${}_{ss}^{{}} {\mathbb{G}}^{a,1}$ matrix by the $\bar{X}_{e}$ vector, and then a linear combination is performed on each vector obtained (linear algebraic form); and (5–6) Apply the Choquet integral on the entries of the vector $\bar{L}$, considering the L-parameter of the L_mδ_-measure equal to − 0.5 (subadditivity) and 0.5 (superadditivity), respectively

Aggregated Objects Type 2 (AO2):

15 $$s(L_{i}^{k} ) = \frac{{\left( {1 - b_{i} } \right)^{\alpha } }}{{\sum\nolimits_{i = 1}^{n} {(1 - b_{i} )^{\alpha } } }},\quad i = 1,2, \ldots ,n$$

where $\alpha \in \left[ {0,\,1} \right]$; and $b_{i}$ constitutes the ith largest of the atom-level descriptors $L_{1}^{k} ,L_{2}^{k} , \ldots ,L_{n}^{k}$. As it can be analyzed, these functions calculate the belonging degree according to the magnitude of the atom-level descriptors, that is, the highest belonging degrees are assigned to the highest atom-level descriptors. The AO1 and AO2 functions are commonly used in the weightings computation for OWA-like operators [73]. However, they satisfy the same mathematical constraints as the singleton measures for the L_mδ_-measure and, thus, they were considered to compute the fuzzy densities in this work. Scheme 2 shows an example of the calculation of these fuzzy 3D-MDs.

**Fig. 1:**
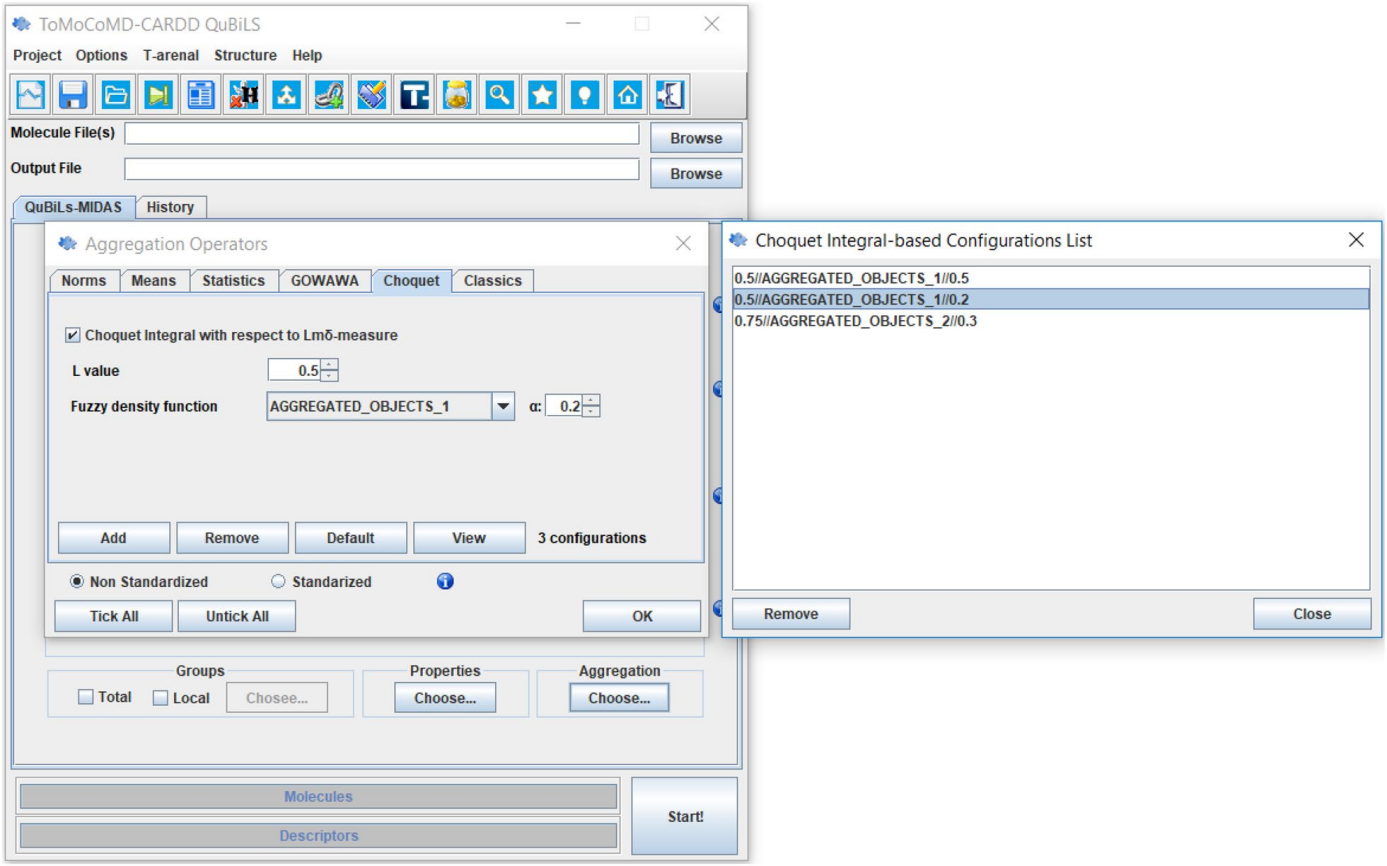
Module built into the QuBiLS-MIDAS software to create different configurations of the Choquet integral-based aggregation operator

Lastly, the computation of these fuzzy global 3D-MDs can be performed through the module built into the QuBiLS-MIDAS software (http://tomocomd.com/qubils-midas) [35]. As it can be seen in Fig. 1, in this module, the value of the L-parameter corresponding to the L_mδ_-measure (see Eq. 1), as well as the singleton measure to be used, can be customized. Several default configurations, determined according to their results in cheminformatics studies, are also provided. These fuzzy 3D-MDs can also be obtained using the distributed computation module coupled to the heterogeneous and non-dedicated T-arenal platform (http://tomocomd.com/t-arenal) [74].

## Performance of the Choquet integral-based fuzzy QuBiLS-MIDAS molecular descriptors

This section is dedicated to demonstrating the feasibility of using the Choquet integral in the fuzzy MDs calculation, by using the QuBiLS-MIDAS 3D-MDs as study case. To this end, models based on the multiple linear regression (MLR) technique were built using the MobyDigs software, which uses the Genetic Algorithm (GA) meta-heuristic as search method [75]. The leave-one-out cross validation $\left( {Q_{loo}^{2} } \right)$ was used as the fitness function. The models retained for further validation were selected according to the best bootstrapping value $\left( {Q_{boot}^{2} } \right)$. All the datasets were optimized with the CORINA software (https://www.mn-am.com/products/corina). From now on, the ‘atomic contributions’ term is only to refer to the atom-level QuBiLS-MIDAS 3D-MDs (LOVIs).

### Configurations for the computation of fuzzy densities

This study is to determine the best configurations for the computation of fuzzy densities. To this end, two project groups with the same configuration of Choquet integral-based fuzzy QuBiLS-MIDAS 3D-MDs were built (see Additional file 2). In both project groups, the value of the *α*-parameter of the functions AO1 (see Eq. 14) or AO2 (see Eq. 15) was varied into the interval $\left[ {0,\,1} \right]$, with a step equal to 0.1. In this way, all the possible configurations were assessed. The value of the L-parameter of the L_mδ_-measure (see Eq. 1) was set to − 0.5 and 0.5, in order to determine the best configurations when a low and a high synergism among the atomic contributions is accounted for. The Cramer’s steroids set [76–80] is the one used to fulfill the goals of this study. This dataset is composed of 31 steroids, so that structures 1–21 and 22–31 belong to the training and test sets, respectively. Compound number 31 is left out at being outlier.

These projects on the steroid’s dataset were calculated (see Additional file 2 for SDF format). For each descriptors matrix obtained (see Additional file 3), models from 1 to 4 variables were created by using the GA-MLR method, in order to predict the binding affinity to the CBG protein [81]. The statistical methods Y-scrambling, bootstrapping, external validation and Fisher function were determined for each model, in order to create a data matrix $M_{n\,x\,k}$, where the $n$ rows and the $k$ columns denote the statistics computed and the configurations to be compared, respectively (see Additional file 4). In this way, the rank (first step of the Friedman test [82]) for each configuration can be computed (see Additional file 5). The configurations selected as the best ones were those with a rank lesser than the difference between the average rank and the standard deviation calculated for a same group.

**Fig. 2:**
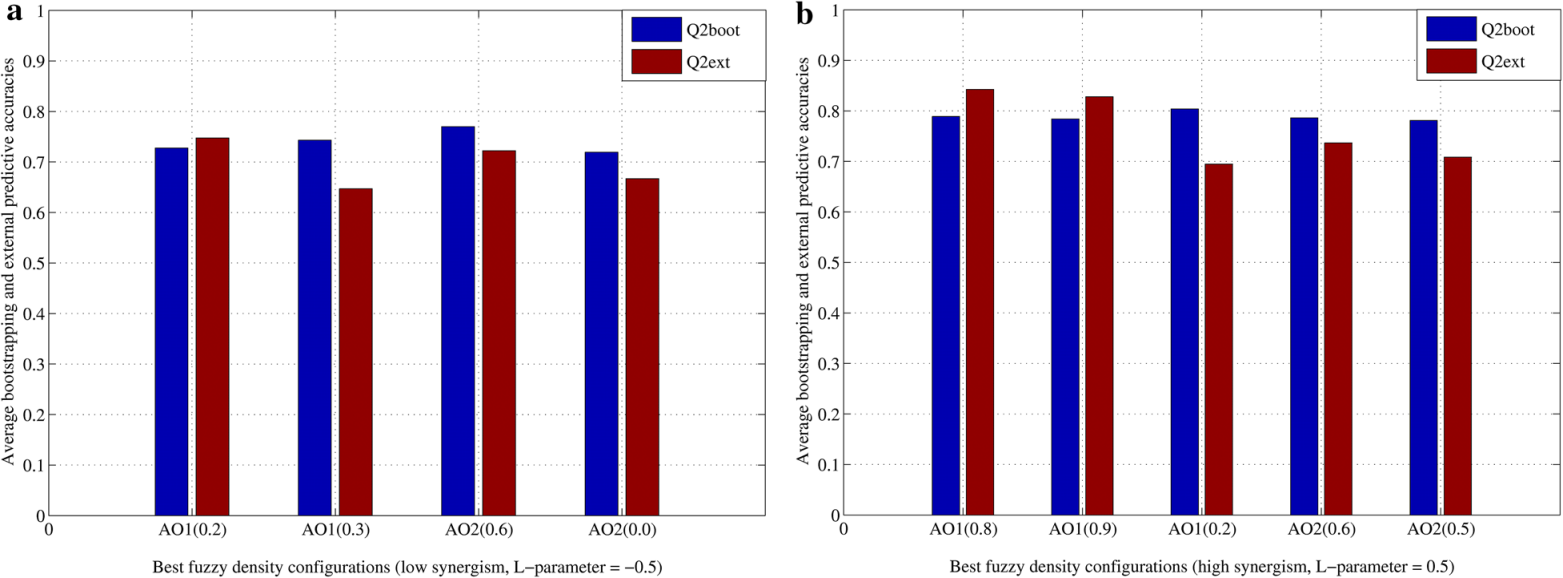
Average bootstrapping accuracy $\left( {Q_{boot}^{2} } \right)$ and average external predictive accuracy $\left( {Q_{ext}^{2} } \right)$ achieved by the best configurations for the computation of fuzzy densities: **a** for a low synergism; **b** for a high synergism

As a result, Fig. 2 shows the average bootstrapping $\left( {Q_{boot}^{2} } \right)$ and external predictive $\left( {Q_{ext}^{2} } \right)$ accuracies corresponding to the best configurations based on functions AO1 (see Eq. 14) and AO2 (see Eq. 15). On one hand, the configurations AO1 $\left( {\alpha = 0.2} \right)$, AO1 $\left( {\alpha = 0.3} \right)$, AO2 $\left( {\alpha = 0.6} \right)$ and AO2 $\left( {\alpha = 0.0} \right)$ are those with the best outcomes when a low synergism is considered during the fuzzy QuBiLS-MIDAS 3D-MDs calculation (see Fig. 2a). On the other hand, at considering a high synergism (see Fig. 2b), the configurations with the best behavior are AO1 $\left( {\alpha = 0.8} \right)$, AO1 $\left( {\alpha = 0.9} \right)$, AO1 $\left( {\alpha = 0.2} \right)$, AO2 $\left( {\alpha = 0.6} \right)$ and AO2 $\left( {\alpha = 0.5} \right)$. In all cases, the average performance achieved by the models is suitable, at presenting $Q_{boot}^{2} > 0.7$ and $Q_{ext}^{2} > 0.6$.

Nonetheless, in a general sense, the configurations obtained for a high synergism (Fig. 2b) present a comparable-to-superior behavior with regard to the configurations obtained for a low synergism (Fig. 2a). These results suggest that the dependency among atomic contributions is an important aspect to consider during the molecular codification with the QuBiLS-MIDAS 3D-MDs. Thus, at least preliminarily, the methodological contribution of this report is justified, in which the 2D/3D-MDs fuzzy calculation from LOVIs/LOEIs is presented. However, more studies must be carried out to prove the feasibility of this fuzzy approach with respect to the traditional approach, where the atom/bond contributions (LOVIs/LOEIs) are considered as non-interrelated values.

### Performance of the Choquet integral-based (fuzzy) QuBiLS-MIDAS descriptors versus norm-, mean- and statistic-based (non-fuzzy) QuBiLS-MIDAS descriptors

In this section, QSAR models based on the QuBiLS-MIDAS 3D-MDs were built to assess the applicability of the Choquet integral in the fuzzy MDs computation from LOVIs/LOEIs. Note that the superiority of the QuBiLS-MIDAS 3D-MDs in modeling tasks was confirmed in [43], where its performance was assessed and compared with regard to 12 methodologies reported in the literature. Therefore, the current study is only devoted to performing an internal analysis among the models built with the fuzzy 3D-MDs (based on Choquet integral) with respect to the models built with the non-fuzzy 3D-MDs (based on traditional operators).

#### Chemical datasets to assess the performance between fuzzy and non-fuzzy QuBiLS-MIDAS descriptors

Eight well-known benchmark datasets were used to carry out this study. These datasets have been widely employed in the literature [83–86], including the analysis to assess the performance of the QuBiLS-MIDAS MDs in QSAR [43]. The datasets are composed of angiotensin converting enzyme (ACE) inhibitors, acetylcholinesterase inhibitors (ACHE), ligands for the benzodiazepine receptor (BZR), cyclooxygenase-2 inhibitors (COX2), dihydrofolate reductase inhibitors (DHFR), inhibitors of glycogen phosphorylase b (GPB), thermolysin inhibitors (THER) and thrombin inhibitors (THR). A description of these datasets is shown in Table 1, whereas Additional file 6 contains the corresponding SDF (Structure Data Format) files.

**Table 1:** Description of the chemical datasets employed to assess the performance of the Choquet integral-based QuBiLS-MIDAS descriptors

	ACE	ACHE	BZR	COX2	DHFR	GPB	THER	THR
Total	114	111	163	322	397	66	76	88
Training	76	74	98	188	237	44	51	59
Test	38	37	49	94	124	22	25	29
Inactive			16	40	36			
Activity	pIC_50_	pIC_50_	pIC_50_	pIC_50_	pIC_50_	p*K*_i_	p*K*_i_	p*K*_i_
Value range	2.1–9.9	4.3–9.5	5.5–8.9	4.0–9.0	3.3–9.8	1.3–6.8	0.5–10.2	4.4–8.5

#### Methodology to assess the performance between fuzzy and non-fuzzy QuBiLS-MIDAS descriptors

Three projects with a same configuration of non-fuzzy QuBiLS-MIDAS 3D-MDs were built, each of them using the norm-, mean-, statistic-based operators, respectively. Two other projects with the same previous configuration were also created but using the Choquet integral to determine the respective fuzzy 3D-MDs. One of the Choquet integral-based projects was designed with the best fuzzy densities obtained for a low synergism among atomic contributions (see Fig. 2a), while the other project was planned with the best fuzzy densities for a high synergism (see Fig. 2b). The L-parameter of the L_mδ_-measure was set to − 0.25, − 0.5 and − 0.75 (subadditivity) in the project considering a low synergism, while the opposite values (superadditivity) were used in the other project. Additional file 6 shows the XML files of the projects described.

These projects on each chemical dataset mentioned above (see Table 1) were computed. Then, the best 1500 variables (MDs) according to the variability criterion [87] were retained by using the IMMAN software [88]. Posteriorly, the GA-MLR procedure was used to build several models for 3, 5 and 7 variables for each operator-type. The best model for each dimension on each dataset was retained (Additional file 7). A pool with the non-fuzzy 3D-MDs and other pool with the fuzzy 3D-MDs included in the best models built on each dataset were created. From these pools, non-fuzzy and fuzzy models for 7 variables were built on each dataset, and the models with the best bootstrapping value were selected as the best ones (Additional file 8). The external validation $\left( {Q_{ext}^{2} } \right)$ statistic parameter was computed for each model developed (see Additional file 7, Additional file 8).

The $Q_{ext}^{2}$ values (predictive abilities) obtained for each chemical dataset were used to establish a comparison and statistical assessment between the models built with the fuzzy and non-fuzzy QuBiLS-MIDAS 3D-MDs, respectively. In this sense, an analysis by means of a boxplot graphic (box-and-whisker graphic) was firstly performed, in order to examine the shape of the distributions of the results achieved. Then, a Wilcoxon signed-rank test [89] was carried out to know whether the predictive abilities achieved by the fuzzy models and the predictive abilities achieved by the non-fuzzy models differ. The SPSS software was used to perform the first analysis mentioned above, while the Keel [90] software was employed to perform the other one. A significance level $\alpha = 0.05$ was accounted for. Note that the ‘fuzzy model’ and ‘non-fuzzy model’ terms are referred to the models built with the fuzzy and non-fuzzy QuBiLS-MIDAS 3D-MDs, respectively.

#### Analysis of the performance achieved by the fuzzy and non-fuzzy QuBiLS-MIDAS descriptors

**Fig. 3:**
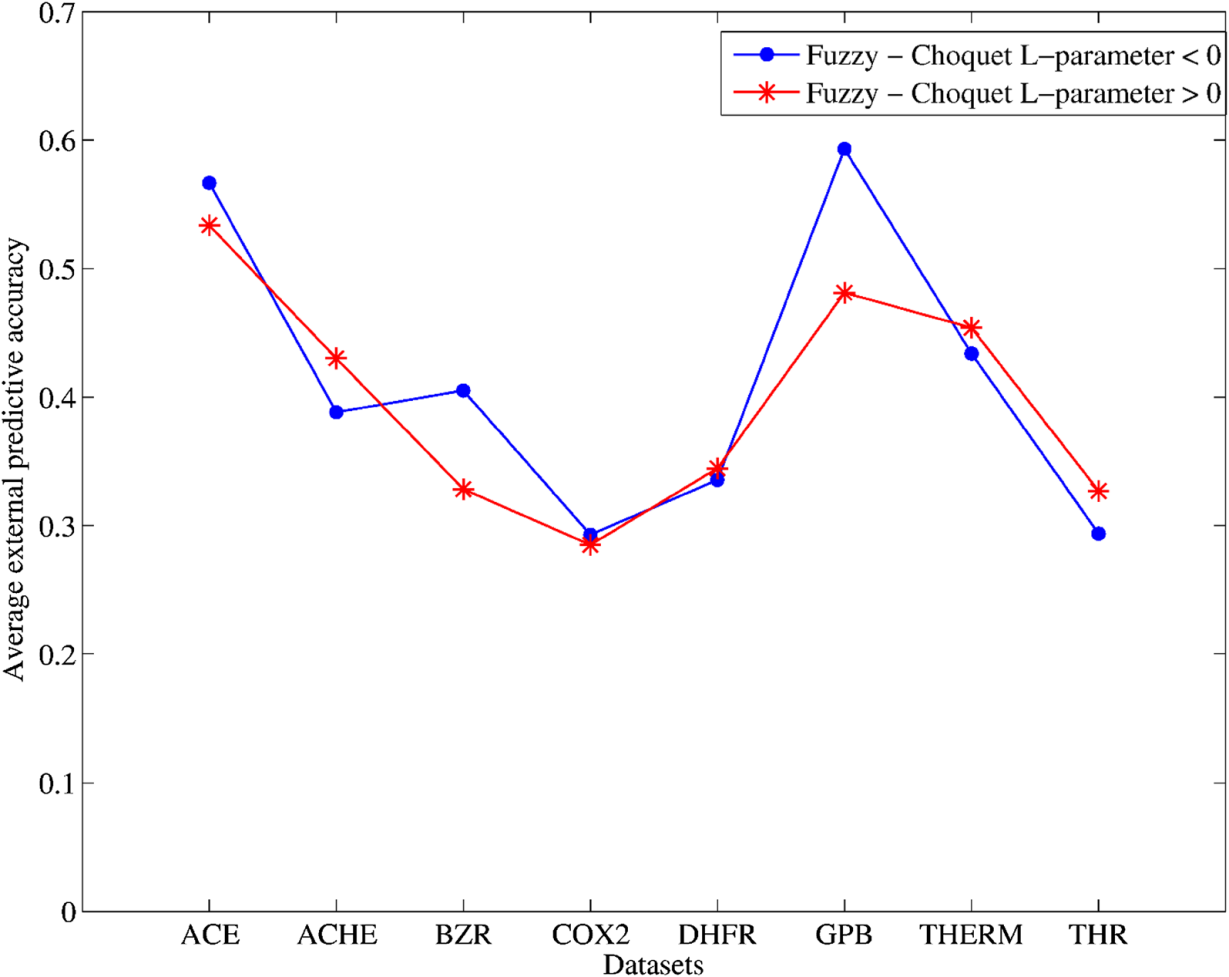
Average external predictive $\left( {Q_{ext}^{2} } \right)$ accuracies corresponding to the models based on the best configurations for the calculation of fuzzy densities, both those determined for a low (L-parameter < 0) and a high (L-parameter > 0) synergism

Figure 3 shows a comparative graphic of the average performance achieved by the models for 3, 5 and 7 variables, built with the fuzzy QuBiLS-MIDAS 3D-MDs, when a low (L-parameter < 0 in Eq. 1) and a high (L-parameter > 0 in Eq. 1) synergism among atomic contributions is accounted for. As it can be seen, the fuzzy 3D-MDs calculated for a low synergism present the best behavior on ACE ($\left( {Q_{ext}^{2} } \right)$ = 0.5667), BZR ($\left( {Q_{ext}^{2} } \right)$ = 0.4052), COX2 ($\left( {Q_{ext}^{2} } \right)$ = 0.2930) and GPB ($\left( {Q_{ext}^{2} } \right)$ = 0.5931) datasets, while the fuzzy 3D-MDs determined for a high synergism present the best behavior on ACHE ($\left( {Q_{ext}^{2} } \right)$ = 0.4303), DHFR ($\left( {Q_{ext}^{2} } \right)$ = 0.3446), THER ($\left( {Q_{ext}^{2} } \right)$ = 0.4541) and THR ($\left( {Q_{ext}^{2} } \right)$ = 0.3270) datasets. So, it is evidenced that fuzzy QuBiLS-MIDAS MDs calculated both for a low and a high synergism contribute to codify useful chemical information, and that their performances depend on the molecular structures under study. Therefore, both types of fuzzy 3D-MDs should be jointly used with the purpose of creating models with better predictive ability.

**Fig. 4:**
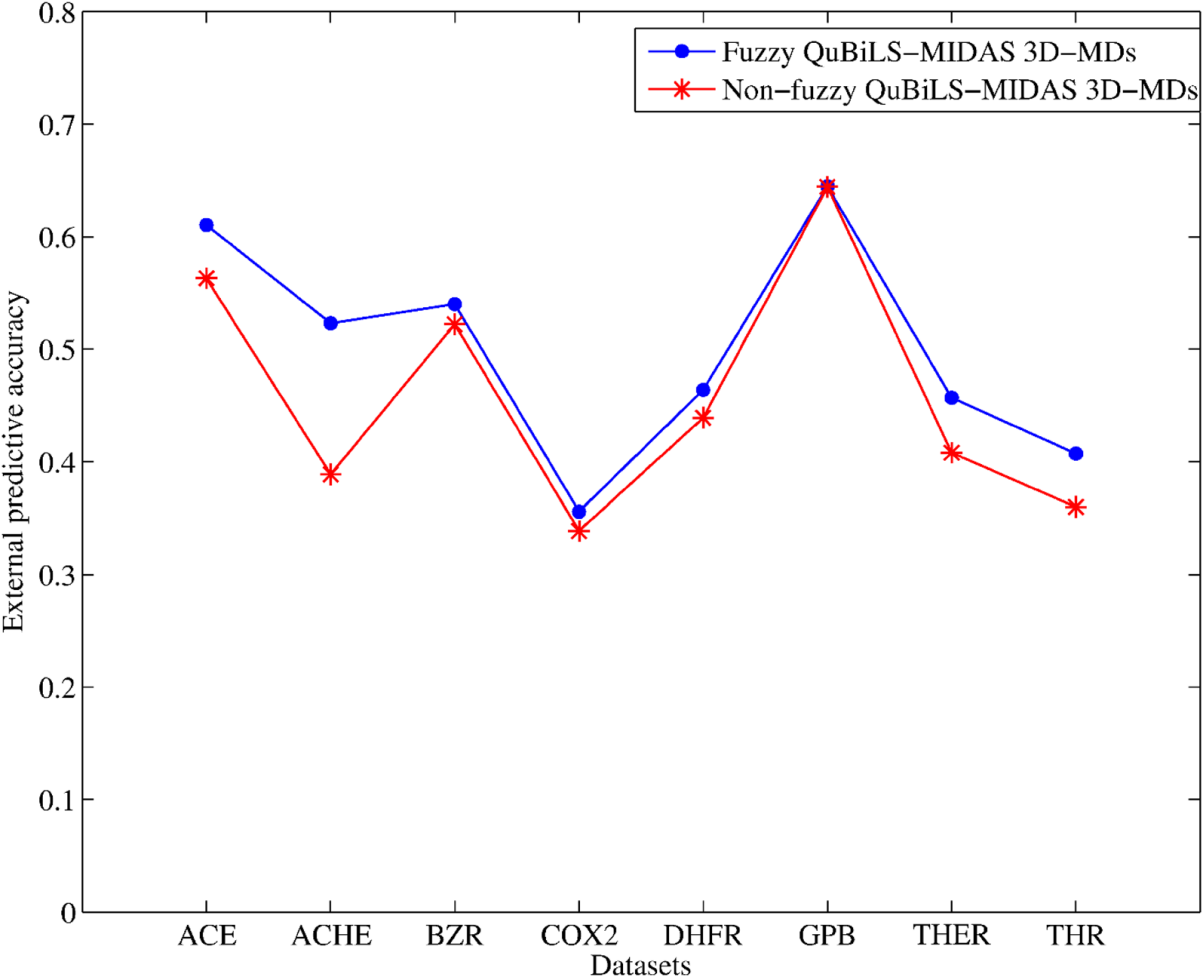
External predictive accuracies $\left( {Q_{ext}^{2} } \right)$ corresponding to the models of 7 variables built with the fuzzy and non-fuzzy molecular descriptors, respectively

In this sense, Fig. 4 shows a plotting of the external predictive ability yielded by the models of 7 variables created with the fuzzy and non-fuzzy QuBiLS-MIDAS 3D-MDs, respectively. The fuzzy MDs determined for a low and a high synergism among atomic contributions were used, while the non-fuzzy MDs used are those computed from the norm-, mean- and statistic-based operators. It can be seen that, in all the chemical datasets employed, the models built with the fuzzy MDs [(ACE, $\left( {Q_{ext}^{2} } \right)$ = 0.6103); (ACHE, $\left( {Q_{ext}^{2} } \right)$ = 0.5231); (BZR, Q_ext_^2^ = 0.5400); (COX2, $\left( {Q_{ext}^{2} } \right)$ = 0.3558); (DHFR, Q_ext_^2^ = 0.4638); (GPB, $\left( {Q_{ext}^{2} } \right)$ = 0.6447); (THER, Q_ext_^2^ = 0.4569); (THR, $\left( {Q_{ext}^{2} } \right)$ = 0.4072)] yield comparable-to-superior performances with regard to the models based on the non-fuzzy MDs [(ACE, $\left( {Q_{ext}^{2} } \right)$ = 0.5629); (ACHE, $\left( {Q_{ext}^{2} } \right)$ = 0.3887); (BZR, $\left( {Q_{ext}^{2} } \right)$ = 0.5222); (COX2, $\left( {Q_{ext}^{2} } \right)$ = 0.3387); (DHFR, $\left( {Q_{ext}^{2} } \right)$ = 0.4390); (GPB, $\left( {Q_{ext}^{2} } \right)$ = 0.6442); (THER, $\left( {Q_{ext}^{2} } \right)$ = 0.4080); (THR, $\left( {Q_{ext}^{2} } \right)$ = 0.3600)]. Thus, it can be stated that MDs with better modeling ability can be calculated using the Choquet integral-based operator, if compared with the MDs computed from the traditional (non-fuzzy) operators.

**Fig. 5:**
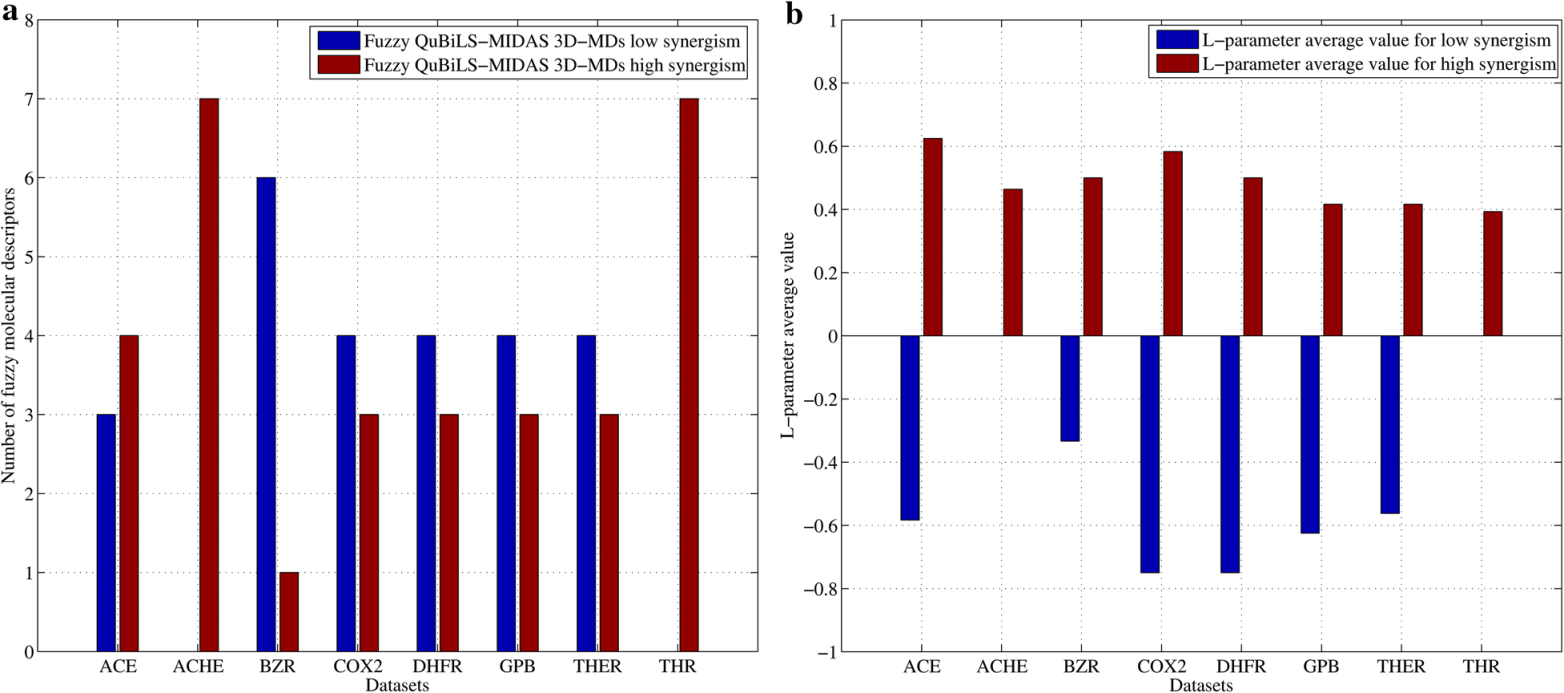
Bar graphics representing: **a** the number of fuzzy QuBiLS-MIDAS 3D-MDs for a low and a high synergism among atomic contributions included into the models built on each dataset used; and **b** the L-parameter average value for the fuzzy QuBiLS-MIDAS 3D-MDs included in the models built on each dataset used

Moreover, Fig. 5a depicts the number of fuzzy QuBiLS-MIDAS 3D-MDs, both for a low and a high synergism among atomic contributions, included in the models built on each dataset. As it can be seen, the fuzzy QuBiLS-MIDAS 3D-MDs determined for a high synergism influenced on the external predictive power of all models developed, being the models corresponding to the ACHE and THR datasets exclusive of these MDs. It can also be noted that the model developed on the ACE dataset presents more QuBiLS-MIDAS 3D-MDs for a high synergism than for a low synergism. A likewise behavior is shown by the models built on the BZR, COX2, DHFR, GPB and THER datasets, but in these cases, there are more fuzzy MDs for a low synergism.

Additionally, Fig. 5b shows the L-parameter average value for the MDs included in the models. In general, it can be seen that, albeit the superadditivity is exclusive for the ACHE and THR datasets, the amount of superadditivity on each dataset is moderate. This behavior can be due to the fact that the datasets used are comprised of congeneric compounds. That is, since the compounds are structurally similar, then MDs computed of additive way, or considering a low synergism among atomic contributions, may be those in achieving better correlations into a QSAR model. This assumption is supported in the average amount of low synergism obtained. As it can be seen, the behavior for a low synergism is from moderate to high, except in the BZR dataset. Thus, at least preliminarily, it can be stated that high amounts of superadditivity will contribute to compute better MDs in non-congeneric datasets than in congeneric datasets.

#### Statistical analysis of the performance achieved by the fuzzy and non-fuzzy QuBiLS-MIDAS descriptors

**Fig. 6:**
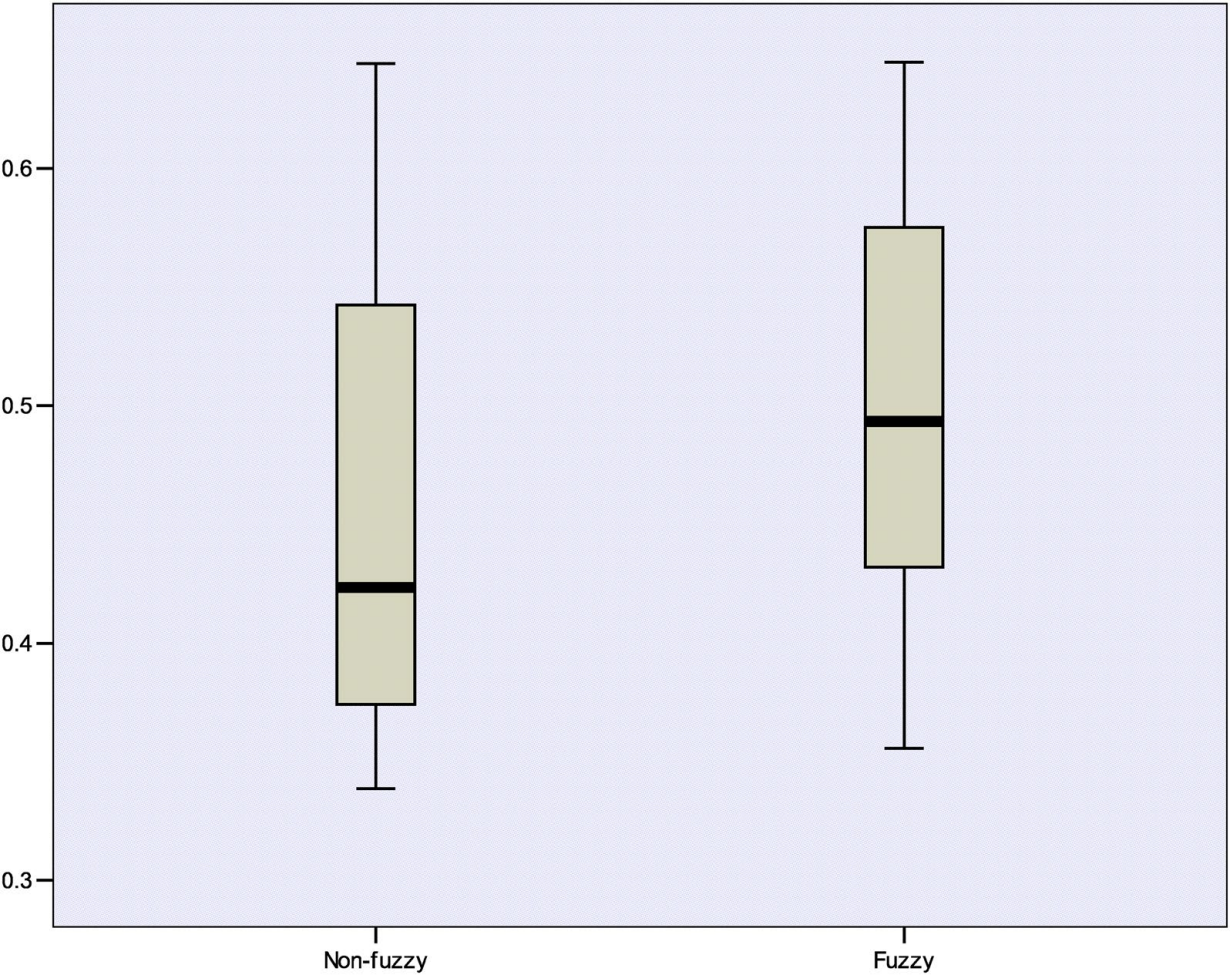
Boxplot graphic corresponding to the external predictive accuracies achieved by the models built with the fuzzy and non-fuzzy QuBiLS-MIDAS 3D-MDs, respectively

To carry out this analysis, the predictive abilities $\left( {Q_{ext}^{2} } \right)$ achieved by the models built with the fuzzy and non-fuzzy QuBiLS-MIDAS 3D-MDs on each dataset were accounted for. Figure 6 shows the boxplot graphic corresponding to the $Q_{ext}^{2}$ values obtained. Additional file 9 shows the descriptive statistics calculated. On one hand, it can be firstly seen that there are not outlier predictive abilities. In addition, it can be seen that the lowest $Q_{ext}^{2}$ attained by the fuzzy models is better than the lowest $Q_{ext}^{2}$ attained by the non-fuzzy models; while the highest outcomes are comparable. It can also be observed that the $Q_{ext}^{2}$ values obtained with the fuzzy models are distributed almost symmetrically (skewness = 0.095); while the $Q_{ext}^{2}$ values obtained with the non-fuzzy models are skewed to the right (skewness = 0.727). These results suggest that the models based on the fuzzy QuBiLS-MIDAS 3D-MDs tend to have a better behavior.

On the other hand, according to the results obtained from the Wilcoxon signed-rank test $\left( {p_{value} \approx 0. 0 0 8} \right)$ (Additional file 10), it can be statistically stated that the $Q_{ext}^{2}$ values achieved by the fuzzy models differ to the ones achieved by the non-fuzzy models. In this sense, if the performances achieved by the models built on each dataset are examined (Additional file 8), it can be appreciated that for the ACHE dataset, the fuzzy model built presents the best progress of all, for a 34.58% of improvement with regard to the non-fuzzy model. Moreover, as for the THR, THERM, ACE, DHFR, COX2 and BZR datasets, the respective fuzzy models improve their predictive abilities a 13.11%, 11.99%, 8.42%, 5.65%, 5.05% and 3.41% with respect to the ones achieved by the non-fuzzy models. Only in the GPB dataset, the improvement of the fuzzy model is insignificant (0.08%). Therefore, in a general sense, it can be concluded that the Choquet integral-based fuzzy 2D/3D-MDs calculation from LOVIs/LOEIs constitutes a prominent alternative to encode relevant chemical information.

## Conclusions

General approaches to compute fuzzy 2D/3D-MDs from the contribution of each atom (LOVIs) or bond (LOEIs) within a molecule were introduced, by using the Choquet integral as fuzzy aggregation operator. The Choquet integral is rather different from the other norm-, mean- and statistic-based (non-fuzzy) operators used to date. It performs a reordering step to fuse according to the magnitude of the criteria and, in addition, it considers the interrelation among criteria by using a fuzzy measure. In this work, the fuzzy L_mδ_-measure was used to compute the importance of the interrelation among atom/bond contributions (LOVIs/LOEIs). In this way, fuzzy descriptors can be derived from traditional or recent descriptors; e.g. fuzzy Balaban-like indices.

The feasibility of this proposal was assessed using the QuBiLS-MIDAS descriptors, by performing modeling studies on eight chemical datasets. It was demonstrated that with the Choquet integral-based descriptors, models with better predictive power can be built, if compared to the models built with the descriptors computed from the other non-fuzzy operators. These outcomes were statistically corroborated using the Wilcoxon signed-rank test. All in all, it can be concluded that the use of the Choquet integral as a fuzzy aggregation operator constitutes a prominent way to extract useful structural information of the molecules and, in this way, enhance the modeling capacity of several existing molecular descriptors in ADME-Tox and pharmacological endpoints.

## Additional files

**Additional file 1.** Definition of the Sugeno Fuzzy λ-measure and the Fuzzy P-measure.

**Additional file 2.** Steroid dataset and projects used to determine the best configurations for the computation of fuzzy densities.

**Additional file 3.** Matrices of descriptors obtained from the calculation of the projects created in Additional file 2 on the Steroid dataset.

**Additional file 4.** Statistical parameters for the models built on each descriptors matrix represented in Additional file 3.

**Additional file 5.** Ranking of the configurations for the computation of fuzzy densities according to the results represented in Additional file 4.

**Additional file 6.** Chemical datasets and projects to assess the performance between fuzzy and non-fuzzy QuBiLS-MIDAS descriptors.

**Additional file 7.** Descriptors included in the best models for 3, 5 and 7 variables built on each chemical dataset shown in Additional file 6.

**Additional file 8.** Best non-fuzzy and fuzzy models of 7 variables built on each chemical dataset shown in Additional file 6.

**Additional file 9.** Descriptive statistics for the external predictions achieved by the best non-fuzzy and fuzzy models of 7 variables represented in Additional file 8.

**Additional file 10.** Results of the Wilcoxon test.
