# Supplementary material for: Choquet integral-based fuzzy molecular characterizations: when global definitions are computed from the dependency among atom/bond contributions (LOVIs/LOEIs)
Source: J Cheminform. 2018 Oct 25;10:51. doi: 10.1186/s13321-018-0306-7 (PMC6755596; doi:10.1186/s13321-018-0306-7)
Supplement: Supplementary file 1 — Additional file 1. Definition of the Sugeno Fuzzy λ-measure and the Fuzzy P-measure. [file 13321_2018_306_MOESM1_ESM.zip › Suppl. Info. 1/Suppl. Info. 1.docx]

### Sugeno Fuzzy λ-measure

A Sugeno fuzzy λ-measure, , on finite set is defined as follows:

(1)

where, and . As it can be observed, the interaction between the criteria sets is represented through the λ parameter. In this way, if , then the function is additive, and thus, the interaction between the criteria sets and is not accounted for. In this case, λ-measure corresponds to the probability measure. Moreover, if , then , and it has multiplicative effect (superadditivity), while if , then , and its effect is substitutive (subadditivity). As is a finite set, then λ-measure satisfies the following definition:

(2)

where , and is a fuzzy density or singleton measure, *i.e.* . This definition is also fulfilled for every . Based on this equation, then the λ-parameter can only be computed when , which is equivalent to resolving the next polynomial equation:

(3)

### Fuzzy P-measure

A fuzzy P-measure, , on finite set is defined as follows:

(4)

where , and is the singleton measure for each . As it can be analyzed, the P-measure determines only the maximum value for any subset and, consequently, it will lead to insensitivity.
