# Supplementary material for: Choquet integral-based fuzzy molecular characterizations: when global definitions are computed from the dependency among atom/bond contributions (LOVIs/LOEIs)
Source: J Cheminform. 2018 Oct 25;10:51. doi: 10.1186/s13321-018-0306-7 (PMC6755596; doi:10.1186/s13321-018-0306-7)
Supplement: Supplementary file 5 — Additional file 5. Ranking of the configurations for the computation of fuzzy densities according to the results represented in Additional file 4. [file 13321_2018_306_MOESM5_ESM.zip › Suppl. Info. 5/1 - Datasets L-measure = -0.5/AO2_STEROIDS_L=-0.5.pdf]

Output tables for 1xN statistical comparisons.

March 4, 2018

1 Average rankings of Friedman test

Average ranks obtained by each method in the Friedman test.

Algorithm	Ranking
AO2(0.0)	5.25
AO2(0.1)	6.125
AO2(0.2)	6.5
AO2(0.3)	7.7
AO2(0.4)	6.3
AO2(0.5)	5.3
AO2(0.6)	5.1
AO2(0.7)	6.05
AO2(0.8)	6.15
AO2(0.9)	6.2
AO2(1.0)	5.325

Table 1: Average Rankings of the algorithms (Friedman)
