# Supplementary material for: Choquet integral-based fuzzy molecular characterizations: when global definitions are computed from the dependency among atom/bond contributions (LOVIs/LOEIs)
Source: J Cheminform. 2018 Oct 25;10:51. doi: 10.1186/s13321-018-0306-7 (PMC6755596; doi:10.1186/s13321-018-0306-7)
Supplement: Supplementary file 9 — Additional file 9. Descriptive statistics for the external predictions achieved by the best non-fuzzy and fuzzy models of 7 variables represented in Additional file 8. [file 13321_2018_306_MOESM9_ESM.zip › Suppl. Info. 9/Descriptive statistics.pdf]

Descriptives

			Statistic	Std. Error
Non-fuzzy	Mean		.457963	.0381014
	95% Confidence Interval for Mean	Lower Bound	.367867	
		Upper Bound	.548058	
	5% Trimmed Mean		.454242	
	Median		.423500	
	Variance		.012	
	Std. Deviation		.1077670	
	Minimum		.3387	
	Maximum		.6442	
	Range		.3055	
	Interquartile Range		.1856	
	Skewness		.727	.752
	Kurtosis		-.667	1.481
Fuzzy	Mean		.500225	.0347981
	95% Confidence Interval for Mean	Lower Bound	.417941	
		Upper Bound	.582509	
	5% Trimmed Mean		.500222	
	Median		.493450	
	Variance		.010	
	Std. Deviation		.0984239	
	Minimum		.3558	
	Maximum		.6447	
	Range		.2889	
	Interquartile Range		.1731	
	Skewness		.095	.752
	Kurtosis		-.879	1.481
