# Supplementary material for: Choquet integral-based fuzzy molecular characterizations: when global definitions are computed from the dependency among atom/bond contributions (LOVIs/LOEIs)
Source: J Cheminform. 2018 Oct 25;10:51. doi: 10.1186/s13321-018-0306-7 (PMC6755596; doi:10.1186/s13321-018-0306-7)
Supplement: Supplementary file 10 — Additional file 10. Results of the Wilcoxon test. [file 13321_2018_306_MOESM10_ESM.zip › Suppl. Info. 10/Wilcoxon.pdf]

Wilcoxon Signed Ranks test.

KEEL non-parametric statistical module

September 28, 2018

1 Detailed results for Non-fuzzy

1.1 Results

VS	R^+	R^-	Exact P-value	Asymptotic P-value
Fuzzy	0.0	36.0	≥ 0.2	1

Table 1: Results obtained by the Wilcoxon test for algorithm Non-fuzzy

1.2 Confidence intervals for Median of diferences

α=0.90	Confidence interval	Exact confidence
Fuzzy	[-0.0761 , -0.01745]	0.92188

Table 2: Confidence intervals for algorithm Non-fuzzy (α=0.90)

α=0.95	Confidence interval	Exact confidence
Fuzzy	[-0.0908 , -0.01265]	0.96094

Table 3: Confidence intervals for algorithm Non-fuzzy (α=0.95)

2 Detailed results for Fuzzy

2.1 Results

VS	R^+	R^-	Exact P-value	Asymptotic P-value
Non-fuzzy	36.0	0.0	0.007812	0.009583

Table 4: Results obtained by the Wilcoxon test for algorithm Fuzzy

2.2 Confidence intervals for Median of diferences

α=0.90	Confidence interval	Exact confidence
Non-fuzzy	[0.01745 , 0.0761]	0.92188

Table 5: Confidence intervals for algorithm Fuzzy (α=0.90)

α=0.95	Confidence interval	Exact confidence
Non-fuzzy	[0.01265 , 0.0908]	0.96094

Table 6: Confidence intervals for algorithm Fuzzy (α=0.95)
