# Supplementary material for: Choquet integral-based fuzzy molecular characterizations: when global definitions are computed from the dependency among atom/bond contributions (LOVIs/LOEIs)
Source: J Cheminform. 2018 Oct 25;10:51. doi: 10.1186/s13321-018-0306-7 (PMC6755596; doi:10.1186/s13321-018-0306-7)
Supplement: Supplementary file 10 — Additional file 10. Results of the Wilcoxon test. [file 13321_2018_306_MOESM10_ESM.zip › Suppl. Info. 10/Wilcoxon_Summary.pdf]

Wilcoxon Signed Ranks test.

KEEL non-parametric statistical module

September 28, 2018

	(1)	(2)
Non-fuzzy (1)	-	0.0
Fuzzy (2)	36.0	-

Table 1: Ranks computed by the Wilcoxon test

	(1)	(2)
Non-fuzzy (1)	-	○
Fuzzy (2)	●	-

Table 2: Summary of the Wilcoxon test. ●= the method in the row improves the method of the column. ○= the method in the column improves the method of the row. Upper diagonal of level significance $\alpha = 0.9$,Lower diagonal level of significance $\alpha = 0.95$

	$\alpha = 0.9$		$\alpha = 0.95$	
Method	$+$	$\pm$	$+$	$\pm$
Non-fuzzy	0	0	0	0
Fuzzy	1	1	1	1

Table 3: Wilcoxon test summary results
